# SGLT2 Inhibitors: From Molecular Mechanisms to Clinical Outcomes in Cardiology and Diabetology

**DOI:** 10.3390/molecules30153112

**Published:** 2025-07-25

**Authors:** Marlena Stielow, Łukasz Fijałkowski, Aidas Alaburda, Grzegorz Grześk, Elżbieta Grześk, Jacek Nowaczyk, Alicja Nowaczyk

**Affiliations:** 1 Department of Organic Chemistry, Faculty of Pharmacy, Collegium Medicum in Bydgoszcz, Nicolaus Copernicus University in Toruń, 2 Jurasza St., 85-089 Bydgoszcz, Poland; marlenastielow@gmail.com (M.S.); l.fijalkowski@cm.umk.pl (Ł.F.); 2 Department of Neurobiology and Biophysics, Institute of Bioscience, Vilnius University, Saulėtekio Ave. 7, LT-10257 Vilnius, Lithuania; aidas.alaburda@gf.vu.lt; 3 Department of Cardiology and Clinical Pharmacology, Faculty of Health Sciences, Collegium Medicum in Bydgoszcz, Nicolaus Copernicus University in Toruń, 87-100 Toruń, Poland; g.grzesk@cm.umk.pl; 4 Department of Paediatrics, Haematology and Oncology, Faculty of Medicine, Collegium Medicum in Bydgoszcz, Nicolaus Copernicus University in Toruń, 9 M. Curie Skłodowska St., 85-094 Bydgoszcz, Poland; ellag@cm.umk.pl; 5 Department of Physical Chemistry and Physicochemistry of Polymers, Faculty of Chemistry, Nicolaus Copernicus University in Toruń, 7 Gagarina St., 87-100 Toruń, Poland; jacek.nowaczyk@umk.pl

**Keywords:** sodium-glucose cotransporter type 2 inhibitors (SGLT2), molecular view of therapeutic mechanisms, clinical research

## Abstract

Studies have shown that sodium-glucose cotransporter type 2 (SGLT2) inhibitors not only help lower blood glucose levels but also offer cardioprotective effects, reduce the progression of heart failure, and may even slow the progression of aortic stenosis. The mechanisms of these beneficial properties are thought to involve multiple pathways, including reducing inflammation, oxidative stress, and improving cellular energy metabolism. Advancing knowledge about the mechanisms of action of these drugs and their effects on the course of the aforementioned diseases has become the subject of intensive clinical and scientific research. This publication aims to provide insight into the role of SGLT2 inhibitors in the context of diabetes mellitus, heart failure and acute coronary syndrome, through clinical analysis, mechanistic insights and comparison of the effects of these drugs.

## 1. Clinical Introduction

Diabetes mellitus, heart failure, and acute coronary syndromes (ACS) are major global health concerns [1] due to their increasing prevalence and significant impact on morbidity and mortality. Diabetes, often referred to as a global epidemic, continues to rise in incidence and contributes to a wide range of complications [2]. Similarly, heart failure remains a leading cause of hospitalization and reduced quality of life, while ACS poses an acute threat due to its sudden onset and potential for severe cardiac damage [3].

These conditions share overlapping pathophysiological mechanisms and frequently coexist, compounding their clinical burden. As a result, there is a growing need for therapeutic strategies that address not only glycemic control but also cardiovascular protection [4].

The subsequent sections explore the function of sodium-glucose co-transporter 2 (SGLT2) inhibitors on metabolic pathways cardiac functions, and vascular remodeling. Special emphasis is given to their mechanisms of action in diabetes, heart failure, and acute coronary syndromes, as well as their broader therapeutic implications.

**Figure 1:**
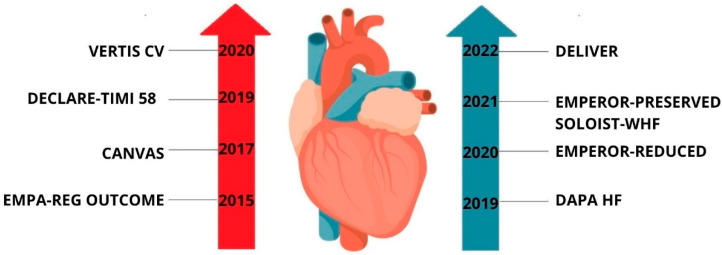
Timeline of major randomized clinical trials evaluating the cardiovascular and heart failure outcomes of SGLT2 inhibitors. Trials in red focus on patients with type 2 diabetes, while those in blue assess heart failure outcomes regardless of diabetes status.

Over the past decade, a series of pivotal clinical trials (summarized in Figure 1) have expanded our understanding of SGLT2 inhibitors beyond glycemic control. Early studies such as EMPA-REG OUTCOME [5], CANVAS [6], DECLARE-TIMI 58 [7], and VERTIS CV [8] demonstrated significant cardiovascular benefits in patients with type 2 diabetes. More recently, trials including DAPA-HF [9], EMPEROR-Reduced [10], EMPEROR-Preserved [11], SOLOIST-WHF [12], and DELIVER [13] have shown that these agents also improve outcomes in heart failure patients, independent of diabetic status. This evolving evidence base has redefined the therapeutic scope of SGLT2 inhibitors, positioning them as multifunctional agents in both cardiology and diabetology [14,15].

SGLT2 inhibitors, commonly referred to as flozins, were originally developed for the treatment of type 2 diabetes mellitus (T2DM). Their primary therapeutic goal was to improve glycemic control by targeting sodium-glucose co-transporter 2 proteins in the kidneys, which are responsible for reabsorbing glucose from the urine into the bloodstream. In T2DM, this reabsorption contributes to hyperglycemia. By inhibiting SGLT2, these drugs promote urinary glucose excretion, thereby lowering blood glucose levels [16].

Beyond their original indication, SGLT2 inhibitors have demonstrated additional metabolic benefits, including modest weight loss and blood pressure reduction. Clinical trials have shown that SGLT2 inhibitors reduce the risk of both microvascular complications, such as diabetic nephropathy [17,18], and macrovascular events, including cardiovascular disease [1,19], ultimately contributing to improved quality of life in patients with T2DM.

Beyond their original indication in T2DM, SGLT2 inhibitors have demonstrated significant benefits in heart failure management [20]. Clinical trials have shown that these agents reduce the risk of hospitalization for heart failure exacerbation [21], improve key cardiac parameters such as left ventricular ejection fraction [22] and stroke volume [23], and reverse adverse cardiac remodeling [24].

Their therapeutic potential now extends to a broader range of conditions. Evidence suggests that SGLT2 inhibitors may reduce cardiovascular risk [25], improve metabolic parameters [17], and even exert neuroprotective effects. In the context of acute coronary syndromes, they have been associated with a reduced incidence of myocardial infarction and stroke, supporting their role as a valuable adjunct in cardiovascular therapy.

This review aims to clarify the molecular mechanisms and systemic effects of SGLT2 inhibitors across diabetes, heart failure, and acute coronary syndromes. By integrating biochemical [26], clinical, and pathophysiological perspectives [27], we seek to highlight the expanding therapeutic relevance of these agents and their significance in shaping future cardiometabolic treatment strategies [28].

## 2. Materials and Methods

Data Selection: The analysis was conducted in accordance with preferred reporting items for systematic review and meta-analyses (PRISMA) guidelines [29].

A literature search was performed using ScienceDirect, Scopus, and PubMed databases to compile a preselected set of publications essential to the topic. A predefined set of keywords was utilized, with “SGLT2” as the primary term. Boolean operator AND was employed to incorporate secondary terms for search refinement, including “bioavailability”, “pharmacokinetics”, “heart failure”, “acute coronary syndrome”, “diabetes mellitus”, “therapeutic efficacy”, and “pharmaceutical innovations”.

The inclusion criteria for this review comprised peer-reviewed articles available in full text, published between 2021 and 2025 in reputable journals. Eligible studies involve human or animal models, articles in English, and research presenting either quantitative or qualitative data pertinent to the topic. Exclusion criteria removed studies that were not relevant to SGLT2 inhibitors applications, non-peer-reviewed sources, publications outside the specified timeframe, and articles with inadequate methodology or ambiguous results. Records duplicated across various databases were consolidated into a single entry.

This methodological framework aimed to ensure a thorough and accurate selection of literature that reflects recent advancements in the field. Ultimately, 109 publications were selected.

## 3. The Molecular Structure of Human SGLT2

**Figure 2:**
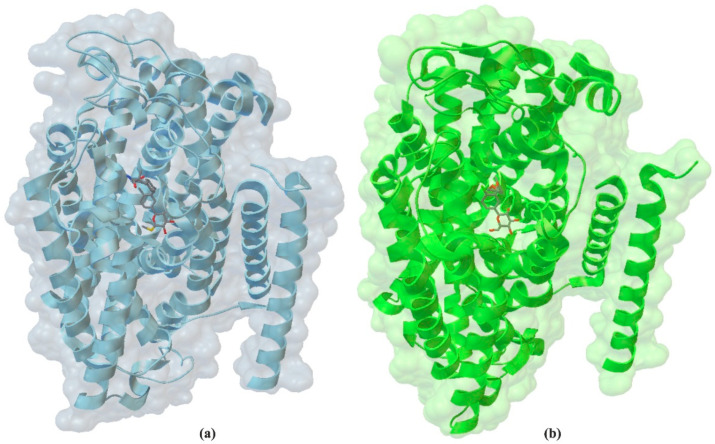
Comparison of the structures of sGLT1 (PDB ID: 7wmv, 3.20 Å) (**a**) and sGLT2 (PDB ID: 7vis, 2.95 Å) (**b**).

In patients with type 2 diabetes mellitus (T2DM), some medications focus on specific kidney transport proteins called SGLTs or sodium-glucose cotransporters. SGLT1, or sodium-glucose co-transporter 1, is a protein encoded in humans by the SLC5A1 gene. It enables the translocation of glucose and galactose across the brush-border membrane of epithelial cells in the small intestine and kidney. This transporter is essential for the absorption of dietary glucose. In contrast to SGLT2 inhibitors, which primarily function by obstructing glucose reabsorption in the kidneys, prospective medicines targeting SGLT1 may restrict the absorption of dietary glucose into the circulation via the intestines. SGLT2 proteins are predominantly located in the kidneys and are involved in the reabsorption of glucose from the primary urine into the circulation. SGLT2 inhibitors facilitate the excretion of surplus glucose in urine by blocking these transporters, hence reducing blood sugar levels without inducing insulin release [30]. A comparison of SGLT1 and SGLT2 proteins is shown in Figure 2.

SGLT2, a trans-membrane protein with 14 helices, has 60% sequence homology to SGLT1. Using expression cloning, researchers identified an accessory membrane-associated protein with a molecular weight of 17 kDa (MAP17). This protein increased SGLT2 activity in RNA-injected Xenopus oocytes by two orders of magnitude. Significant stimulation of SGLT2 activity also occurred in opossum kidney cells co-transfected with SGLT2 and MAP17. It should be emphasized that transfection with MAP17 did not change the amount of SGLT2 protein on the cell surface in either cell type. The physiological significance of the MAP17–SGLT2 interaction was confirmed in a study involving a cohort of 60 individuals with familial renal glucosuria, as reported by Coady et al. [31].

## 4. SGLT2 Inhibitor Mechanisms of Action in Diabetic Mellitus

The physiology of glucose in the body includes its key role as the main source of energy for cellular functions and regulatory processes, ensuring the maintenance of metabolic homeostasis [32,33]. After carbohydrate ingestion, glucose is released into the digestive system and absorbed into the bloodstream. Next, glucose flows via the circulation to numerous tissues and organs, where it serves as a primary energy source [34]. In muscles, glucose can be used to produce the energy needed for muscle contractions. However, in hepatocytes, glucose can be processed and stored as glycogen, which can be used as fuel at a later time. The liver plays a key role in regulating blood glucose levels by storing and releasing glucose in response to the changing energy needs of the body [35].

Various hormones, such as insulin and glucagon, carefully regulate blood glucose levels. Insulin, released by pancreatic β cells in response to rising blood glucose levels after a meal, contributes to glucose absorption and promotes glycogen accumulation in muscles and liver [36]. On the other hand, glucagon, secreted by the pancreatic α cells in response to a decrease in blood glucose levels, stimulates the release of glucose from the liver through glycogenolysis [37]. Thus, blood glucose regulation is a complicated mechanism that maintains steady blood glucose levels required for the body’s normal operation while simultaneously ensuring the availability of glucose as the primary energy source for cells [38].

In the renal tubules, the filtration of glucose from the blood into the nephron tubules occurs in a structure called the renal glomerulus. The main goal of this process is to separate nutrients, such as glucose, from waste products of metabolism, which will be excreted from the body along with urine. After being filtered through the renal glomerulus, glucose, along with other substances, is retained in the filtrate, known as glomerular filtrate [39]. The filtered glucose is then sent to the tubules of the nephron to be reabsorbed into the bloodstream. The proximal tubule of the nephron is where this process takes place. SGLT2 is essential to this process [40]. SGLT2, a transporter primarily located in the S1 segment of the proximal tubule of the nephron. This transporter is specialized in reabsorbing glucose from the glomerular filtrate back into the epithelial cells of the renal tubule. Approximately 90% of the glucose that has been filtered in the renal glomerulus is reabsorbed through SGLT2. The remaining portion of glucose (about 10%) and other substances that were not reabsorbed by SGLT2 continue their journey through the nephron tubule [41]. In the further segments of the proximal tubule, glucose that was not reabsorbed by SGLT2 can be reabsorbed by another transporter, SGLT1, which also participates in the process of glucose reabsorption in the kidneys [42]. SGLT2 is crucial in modulating glucose levels in the body by governing its reabsorption from urine into the circulation inside the kidneys. SGLT2 inhibitors function by obstructing the activity of SGLT2, resulting in heightened glucose excretion in urine and reduced blood glucose levels, which is beneficial for diabetes management [25].

**Figure 3:**
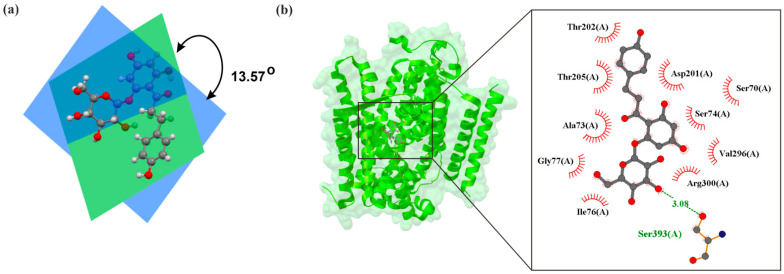
(**a**) Diagram of crystallographic structures of phlorizin (CCDC: CEWWAC01), with indication of the angle between planes defined by phenolic ring (green) and glycosidic (blue), (**b**) X-ray structure of human SGLT2–MAP17 complex bound with phlorizin in the inward conformation crystal with the grid box showing the ligand-binding site with phlorizin (PDB ID: 8hin, 3.30 Å) [43]. The red circles and ellipses identify equivalent residua (in 3D superposition of structures).

Initially, research on SGLT2 inhibitors focused on a natural compound known as phlorizin. It is an O-glucoside (Figure 2a) that is hydrolyzed by β-glucosidase in the intestines. The structure of the human hSGLT2–MAP17 complex with phlorizin, as determined by Hiraizumi et al. [43] using cryo-electron microscopy, is shown in Figure 3. Phlorizin interacts with hSGLT2–MAP17 in an internal configuration, as depicted in Figure 2b. The interaction between phlorizin and hSGLT2 exhibits biphasic kinetics, suggesting that phlorizin binds alternately to the extracellular and intracellular domains. Na^+^-bound structures oriented outward and unbound structures directed inward of hSGLT2–MAP17 suggest that the MAP17-binding domain of the bundle functions as a scaffold, with the hash domain rotating around the Na^+^-binding site. Na^+^ binding stabilizes the outward-facing conformation, whereas its release promotes the transition to an inward-open conformation, demonstrating Na^+^’s role in the symport process.

However, due to the poor metabolic stability of phlorizin, its oral use was difficult. In order to replace phlorizin, compounds with better metabolic stability began to be sought. As a result, N- or C-glucosides emerged, which exhibited significantly better metabolic stability. Among these compounds were canagliflozin (CANA), dapagliflozin (DAPA), and empagliflozin (EMPA), which have been approved as drugs in the United States, Japan, and many other countries [43,44]. The selection of these agents in our review was based on their widespread clinical use and inclusion in major cardiovascular outcome trials. More recently, additional SGLT2 inhibitors such as bexagliflozin (approved in the USA in 2023) and enavogliflozin (approved in South Korea in 2022) have entered clinical use, reflecting the expanding therapeutic landscape of this drug class. SGLT2 inhibitors, such as DAPA, EMPA, and CANA, exert their effect by selectively blocking the action of SGLT2 in the renal tubules [45].

**Figure 4:**
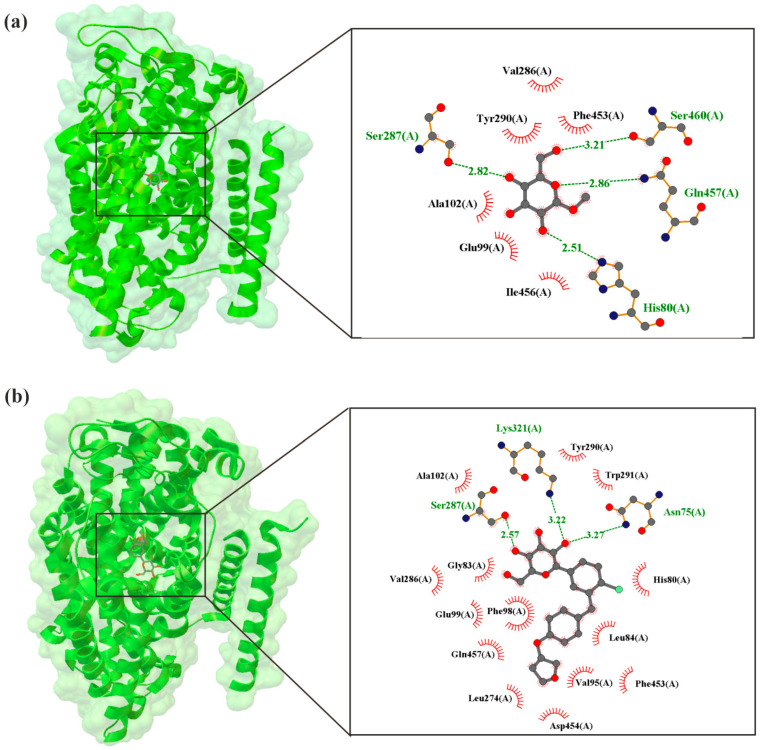
X-ray structure of the human SGLT2–MAP17 complex bound with substrate α-methyl-D-galactopyranoside (AMG) in the occluded conformation crystal (**a**) with the grid box showing the ligand-binding site with AMG (PDB ID: 7ynjj, 3.33 Å) [47], (**b**) with the grid box showing the ligand-binding site with EMPA (PDB ID: 7vsi, 3.33 Å) [46]. The red circles and ellipses identify equivalent residua (in 3D superposition of structures).

As illustrated in Figure 4, Niu et al. [46], using cryo-electron microscopy, present the structures of human hSGLT2–MAP17 complexed with synthetic inhibitors. It shows the EMPA molecule interacts with the SGLT2 protein and is surrounded by TM1, TM2, TM6, and TM10 [46]. EMPA binds to the sugar–substrate binding site and the outer atrium, effectively blocking hSGLT2 in an outward-open conformation, which inhibits the transport cycle.

**Figure 5:**
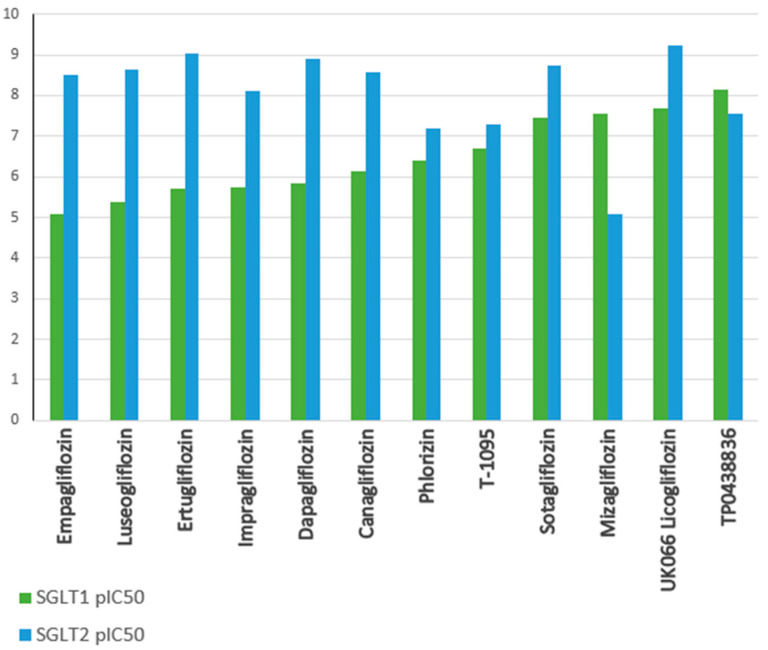
PIC_50_ values of selected SGLT1, SGLT2, and dual SGLT1/SGLT2 inhibitors needed to inhibit a biological process or component by 50% in vitro.

SGLT2 inhibitors prevent glucose from being reabsorbed from the kidney’s filtering system back into the blood, which results in more glucose being removed in urine and lowers blood glucose levels. This mechanism underlies the effectiveness of these drugs in glycemic control in individuals with T2DM [48]. In addition, inhibition of renal glucose reabsorption by SGLT2 inhibitors may lead to further beneficial health effects. For example, increased urinary glucose excretion translates into a decrease in caloric glucose absorption, which may contribute to weight loss in overweight or obese patients [49]. Furthermore, the loss of glucose through the urine results in increased water excretion, leading to a mild diuretic effect and a reduction in body fluid volume, which may further lower blood pressure. Therefore, SGLT2 inhibitors may be particularly beneficial in patients with hypertension [50]. As a result, the mechanism of action of SGLT2 inhibitors includes not only glycemic control by inhibiting glucose reabsorption in the kidneys but may also bring additional health benefits, such as weight reduction, lower blood pressure, and reduced accumulation of visceral fat, making them promising drugs in the treatment of T2DM and its complications [51]. In recent years, it has also been noted that inhibition of SGLT1 in the gastrointestinal tract affects post-meal glucose regulation and the secretion of gastrointestinal hormones. Therefore, dual SGLT1 and SGLT2 inhibitors, such as sotagliflozin (SOTA) and LX-2761, are currently being investigated as potential drugs for the treatment of diabetes (Figure 5) [43,46,52].

Beyond their therapeutic efficacy in glycemic control and metabolic regulation, the structural design of SGLT2 inhibitors carries broader implications. The molecular features that enhance their selectivity and metabolic stability, such as the C-glucoside scaffold [53] and halogenated aromatic rings, not only optimize renal glucose transport inhibition but also contribute to environmental persistence [54]. These compounds, once excreted, may resist biodegradation in aquatic ecosystems, raising concerns about pharmaceutical pollution [55]. Although environmental aspects of pharmaceuticals are increasingly recognized in water pollution screening studies, they remain underrepresented in other branches of drug-focused literature.

The multifaceted nature of SGLT2 inhibitors extends beyond diabetes management. In the following section, we explore their mechanisms of action in heart failure, where metabolic modulation and anti-inflammatory effects play a pivotal role in improving cardiac outcomes.

## 5. Mechanisms of Action of SGLT2 Inhibitors in Heart Failure

The therapeutic effects of SGLT2 inhibitors in heart failure have attracted growing attention in recent years. Originally developed for glycemic control, these drugs have shown benefits that go far beyond lowering blood sugar. Their mechanisms of action in heart failure are complex and multifaceted, involving changes in cardiac metabolism, reduction of fibrosis, modulation of inflammation, and improvement of oxidative balance. In the following subsections, we discuss these effects in more detail.

### 5.1. Modulation of Cardiac Energy Metabolism

In the pathogenesis of heart failure, the role of glucose as an energy substrate becomes particularly important. Under normal conditions, the heart primarily relies on fatty acid oxidation (FAO) for ATP production. However, FAO is oxygen-intensive, and in the failing heart, this metabolic pathway becomes less efficient [56]. As a result of chronic metabolic stress, cardiomyocytes shift toward increased reliance on fatty acids, a phenomenon known as the Randle cycle [57,58]. This shift, although compensatory at first, can lead to lipid accumulation and metabolic dysfunction, further impairing cardiac performance [59].

In this context, SGLT2 inhibitors may help restore metabolic flexibility. These drugs promote a shift toward glucose and ketone body utilization, which are more oxygen-efficient fuels. Glucose oxidation yields more ATP per molecule of oxygen compared to fatty acids, which may help reduce the metabolic burden on the failing heart [60]. Moreover, this shift is supported by transcriptional changes in key metabolic enzymes and improved mitochondrial efficiency [61].

By enhancing the availability of glucose and ketones, SGLT2 inhibitors may alleviate energy deficits in cardiomyocytes, reduce inflammation, and improve mitochondrial function [62]. These effects contribute to better cardiac energetics and may explain part of the observed clinical benefits in heart failure patients.

### 5.2. Antifibrotic Effects

Fibrosis is a key pathological feature in heart failure, contributing to myocardial stiffness, impaired contractility, and progressive cardiac dysfunction. High glucose levels, oxidative stress, and chronic inflammation all promote fibroblast activation and excessive deposition of extracellular matrix components, particularly collagen [63].

SGLT2 inhibitors appear to counteract these processes through several mechanisms. By lowering blood glucose levels, they reduce the formation of advanced glycation end products (AGEs), which are known to stimulate fibrotic signaling pathways. In addition, these inhibitors may indirectly suppress the activity of pro-fibrotic mediators such as transforming growth factor-β (TGF-β) and matrix metalloproteinases, which are involved in extracellular matrix remodeling [64].

Experimental studies have shown that treatment with SGLT2 inhibitors leads to a reduction in myocardial collagen content and improved ventricular compliance [65]. These antifibrotic effects may be particularly relevant in patients with heart failure with preserved ejection fraction (HFpEF), where diastolic dysfunction is often driven by myocardial stiffening.

Altogether, the ability of SGLT2 inhibitors to limit fibrotic remodeling adds another layer to their cardioprotective profile and may help explain their clinical benefits in heart failure.

### 5.3. Anti-Inflammatory Mechanisms

Chronic inflammation plays a central role in the progression of heart failure. It contributes to myocardial injury, remodeling, and fibrosis, and is often sustained by metabolic disturbances such as hyperglycemia and insulin resistance. Inflammatory cytokines like IL-1β, TNF-α, and IL-6 are elevated in heart failure and are associated with worse outcomes [63].

SGLT2 inhibitors appear to exert anti-inflammatory effects through several pathways. One of the key mechanisms involves the inhibition of the NF-κB signaling pathway, which regulates the expression of many pro-inflammatory genes [66]. By reducing glucose toxicity and oxidative stress, these inhibitors may also lower the activation of immune cells such as macrophages, which are known to adopt a pro-inflammatory phenotype in the diabetic and failing heart [63].

Experimental studies have shown that SGLT2 inhibitors reduce circulating levels of inflammatory markers and attenuate immune cell infiltration in cardiac tissue [67]. These effects may help to stabilize the myocardial environment, reduce tissue damage, and slow disease progression.

Taken together, the anti-inflammatory properties of SGLT2 inhibitors contribute to their cardioprotective profile and may be particularly relevant in patients with heart failure and metabolic comorbidities.

### 5.4. Antioxidant Properties

Oxidative stress is another important contributor to the progression of heart failure. It results from an imbalance between the production of reactive oxygen species (ROS) and the antioxidant defense systems of the body. In the failing heart, excessive ROS can damage cellular structures, impair mitochondrial function, and activate pro-inflammatory and pro-fibrotic pathways [68].

It has been demonstrated that SGLT2 inhibitors can mitigate oxidative stress through a variety of mechanisms. One of the primary impacts is an increase in nitric oxide (NO) bioavailability, which helps to maintain vascular tone and minimize endothelial dysfunction. Additionally, SGLT2 inhibitors may lower ROS production by blocking NADPH oxidase action and raising the efficiency of mitochondria [69].

These antioxidant effects are not limited to the vasculature. In cardiac tissue, SGLT2 inhibitors may protect cardiomyocytes from oxidative injury and improve redox balance, which is essential for maintaining contractile function and preventing further damage [68].

Summing up, the ability of SGLT2 inhibitors to reduce oxidative stress adds to their multifaceted cardioprotective profile and may be particularly beneficial in patients with heart failure and comorbid metabolic disorders.

### 5.5. Hemodynamic and Neurohormonal Modulation

Although SGLT2 inhibitors were not initially developed as antihypertensive agents, their effects on blood pressure and volume status are well documented. By promoting glycosuria and natriuresis, these drugs lead to a mild diuretic effect, which reduces plasma volume and preload. This contributes to a decrease in cardiac workload and may help reduce symptoms in patients with heart failure [41].

In addition to their hemodynamic effects, SGLT2 inhibitors appear to modulate neurohormonal systems involved in heart failure progression. They may reduce the activation of the renin–angiotensin–aldosterone system (RAAS), which plays a vital role in sodium retention, vasoconstriction, and cardiac remodeling [27]. By attenuating RAAS activity, these inhibitors may help prevent further deterioration of cardiac function.

The inclusion of SGLT2 inhibitors in a thorough strategy for managing heart failure is justified by the combined effects of volume reduction, blood pressure lowering, and modulation of neurohormones [70]. This is particularly the case for patients who have volume overload and neurohormonal dysregulation.

## 6. Mechanisms of Action of SGLT2 Inhibitors in Acute Coronary Syndrome

Endothelial dysfunction plays a key role in the pathogenesis of acute coronary syndrome (ACS), an emergency condition associated with myocardial ischemia. It is a process that involves various changes in the function and structure of the endothelium, leading to disturbances in the reactivity of coronary vessels and resulting in inflammatory processes and the formation of clots [71]. An excessive amount of reactive oxygen species (ROS) is responsible for the occurrence of endothelial dysfunction. ROS are responsible for activating signaling pathways, including ERK, JNK, and p38 MAPKs, as well as increasing the expression of NF-κB [72]. The endothelium plays a crucial role in regulating vascular tone through the production of various substances, including nitric oxide (NO), which is an important vasodilatory factor [73,74]. In ACS, endothelial dysfunction can lead to a decrease in NO production or a reduction in its action, which results in impaired coronary vascular reactivity. Vasoconstriction can limit blood flow to the heart muscle, increasing the risk of ischemia and the occurrence of symptoms of CAD, such as chest pain [75]. Endothelial dysfunction can lead to increased secretion of pro-inflammatory cytokines and adhesion of inflammatory cells to the endothelium. These inflammatory processes can contribute to the damage of coronary vessels through the activation of the coagulation system, increased platelet adhesion, and the induction of pro-inflammatory processes within the endothelium, which in turn promote thrombus formation and vessel occlusion [76]. Furthermore, the endothelium is responsible for the inhibition of platelet aggregation and leukocyte adhesion, both of which contribute to the preservation of homeostasis in blood vessels. It is possible for endothelial dysfunction to result in a decrease in this anti-aggregatory activity, which in turn raises the probability of thrombus formation and the blockage of coronary arteries [77]. Endothelial dysfunction is a crucial element in the pathophysiology of coronary artery disease (CAD). This dysfunction leads to decreased vascular reactivity, inflammatory processes, and diminished anti-aggregatory function, all of which raise the likelihood of myocardial ischemia and the emergence of coronary artery disease (CAD) symptoms [78].

Anti-inflammatory effects observed as an additional benefit of SGLT2 inhibitors may reduce coronary vessel damage in coronary artery disease (CAD). As a result of the decrease in oxidative stress, the suppression of pro-inflammatory cytokine release, and the enhancement of endothelial function, SGLT2 inhibitors have the potential to reduce inflammation in coronary veins and minimize the risk of clotting [79]. Oxidative stress occurs when the production of reactive oxygen species (ROS) exceeds the body’s antioxidant defense capacity, leading to cellular and tissue damage, including injury to blood vessels [80].

SGLT2 inhibitors, such as EMPA, have demonstrated their ability to increase nitric oxide (NO) synthesis by inhibiting ROS production in endothelial cells. Nitric oxide plays an important role in regulating inflammation and vascular reactivity. It is produced by the endothelium and is an important vasodilator [81,82]. Endothelial function is improved by SGLT2 inhibitors since they increase the generation of nitric oxide. SGLT2 inhibitors prevent the generation of ROS in endothelial cells, independent of glucose levels. EMPA decreases ROS generation in both venous and arterial endothelial cells, which helps reduce vascular inflammation. EMPA enhances NO bioavailability in endothelial cells, allowing neighboring cardiomyocytes (CM) to contract and relax more effectively via the endothelium–NO route. SGLT2 inhibitors additionally decrease ROS levels via blocking the sodium–hydrogen exchanger (NHE), NADPH oxidase (NOX), and mitochondrial fission and fusion [72,83,84].

The primary purpose of SGLT2 inhibitors is to decrease glucose reabsorption in the kidneys, hence increasing urine glucose excretion. Blood glucose levels are lowered, which reduces the oxidative damage caused by glucose metabolism [85]. By inhibiting the function of the RAAS system, which plays a role in the regulation of blood pressure and fluid balance, SGLT2 inhibitors have the potential to reduce the oxidative stress that is linked with inflammatory processes and coronary vascular reactivity [86]. Furthermore, these inhibitors diminish the release of pro-inflammatory cytokines, including interleukins (IL) and tumor necrosis factor-alpha (TNF-α), by obstructing signaling pathways associated with their secretion, notably the NF-κB pathway [87], and by modifying the gut microbiota composition. Research indicates that EMPA medication results in alterations to the gut flora. A rise in Bacteroidetes bacteria and a concurrent decline in Firmicutes were noted following the administration of this medication. These alterations in the gut microbiota’s makeup are linked to decreased insulin resistance, obesity, and the activation of pro-inflammatory and profibrotic pathways [88].

Recent findings have identified SGLT2 inhibitors as the first class of drugs with direct endothelial-protective properties regardless of their glycemic effects. Viggiano et al. have demonstrated that under diabetic and stress conditions, SGLT2 is abnormally expressed by endothelial cells, which causes intracellular sodium and glucose buildup and endothelial dysfunction. SGLT2 inhibitors counteract this by blocking SGLT2 activity in these cells, protecting the integrity of the endothelium and the operation of the mitochondria [89]. This mechanism may explain why SGLT2 inhibitors work so well in people who do not have diabetes, and it supports their role in avoiding vascular complications in addition to controlling their blood sugar levels.

The notion that hyperglycemia is the only cause of diabetes complications is being increasingly contested. Viggiano (2023) hypothesised that diabetic nephropathy and vascular impairment may endure despite good glycaemic regulation, indicating the potential influence of supplementary metabolic and hormonal variables [90]. These observations underscore the many advantages of SGLT2 inhibitors and their potential use in wider treatment scenarios, including age-related vascular dysfunction.

**Figure 6:**
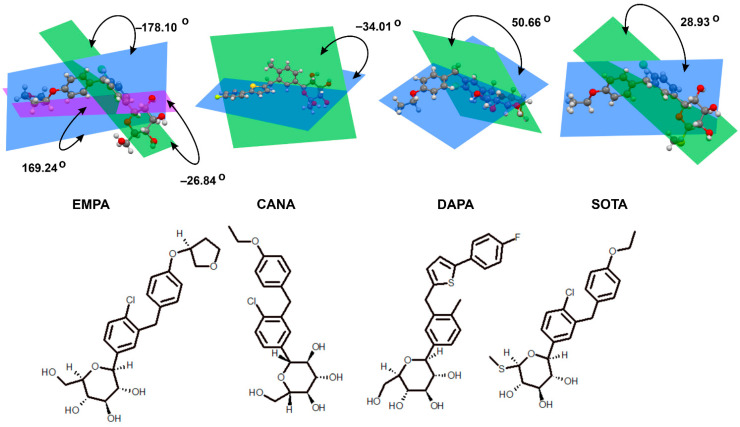
Diagram of crystallographic structures of empagliflozin (EMPA; CCDC: NIQBIB01), dapagliflozin (DAPA; CCDC: CIMNUJ), canagliflozin (CANA; CCDC: XIKWOH), and sotagliflozin (SOTA; CCDC: TEJGAT). The planes shown in the structures are defined by respective rings, aromatic ring (green) and glycosidic ring (blue) and allow to determine specific interring angle.

SGLT2 inhibitors act in the renal tubules to inhibit glucose reabsorption, leading to increased urinary glucose elimination. This mechanism also affects sodium reabsorption, as both glucose and sodium are transported by the same transporter (SGLT2) [86]. SGLT2 inhibitors decrease the activity of Na^+^/H^+^ (NHE) transporters, including NHE3, resulting in increased natriuresis, or urine sodium excretion. EMPA, DAPA, and CANA are commonly used medications in clinical practice. EMPA is the most selective in its effect on the SGLT2 transporter when compared to SGLT1, with a more than 2500-fold increase in activity against SGLT2 versus SGLT1. For instance, CANA exhibits reduced selectivity for SGLT2, with an approximately 155-fold increase relative to SGLT1 (Figure 6) [91].

SGLT2 inhibitors contribute to lowering blood pressure primarily through their effects on renal Na^+^ and glucose reabsorption. By inhibiting the SGLT2 transporter in the renal tubules, these drugs increase urinary excretion of both glucose and sodium (natriuresis) [92], leading to a reduction in circulating blood volume and subsequently lowering blood pressure [41,86,91]. This decrease in blood pressure reduces the workload on the heart, as it has to pump a smaller volume of blood against less vascular resistance [93,94]. In heart failure, this mechanism is particularly beneficial, as it alleviates cardiac strain and supports improved myocardial efficiency [95].

In the context of acute coronary syndrome (ACS), where myocardial hypoxia and increased cardiac workload are critical concerns, the reduction in blood pressure and circulating volume provided by SGLT2 inhibitors helps maintain adequate coronary perfusion [96]. Lowered blood pressure reduces the burden on coronary vessels, facilitating better blood flow and potentially aiding in rapid reperfusion during acute myocardial infarction (AMI), which is essential for minimizing myocardial damage [97]. Thus, by decreasing cardiac workload and enhancing coronary circulation, SGLT2 inhibitors offer significant vascular benefits in ACS.

As a result, the inhibition of sodium reabsorption by SGLT2 inhibitors can contribute to a reduction in circulating blood volume, lower blood pressure, decreased cardiac workload, and improved blood flow in the coronary vessels. These effects collectively help reduce the risk of cardiovascular complications and improve the overall course of the disease [91].

## 7. Comparison of the Mechanisms of Action of SGLT2 Inhibitors in Diabetes, Heart Failure, and Acute Coronary Syndrome

The basic mechanisms of action, differences and similarities in action, the therapeutic synergy occurring among the three disease entities discussed, and the prospects for further research are presented in Table 1.

The similarities in the action of these drugs result from their impact on blood glucose levels, blood pressure, and heart function. At the same time, the differences in their action reflect the specifics of each of these diseases. In diabetes, the primary goal of therapy is to control blood glucose levels, which is achieved through increased glucose excretion in the urine [94]. In heart failure, SGLT2 inhibitors contribute to decreasing the burden on the heart and assist cardiomyocytes in transitioning to more efficient energy sources [98]. In the case of ACS, these drugs can improve endothelial function, reduce oxidative stress, and decrease the risk of thrombosis. The therapeutic synergy between different diseases results from the multifaceted action of SGLT2 inhibitors [99]. In the case of coexisting diabetes with heart failure or ACS, SGLT2 inhibitors can reduce the risk of cardiovascular complications, lower blood pressure, and reduce inflammation [100].

## 8. Neuroprotective Potential of SGLT2 Inhibitors: Mechanisms and Therapeutic Implications for Central Nervous System Disorders

Recent studies emphasize the possible neuroprotective properties of SGLT2 inhibitors on the central nervous system (CNS). In addition to their known function in glucose regulation, these inhibitors seem to reduce oxidative stress, neuroinflammation, and amyloid deposition, all significant contributors to neurodegeneration [101]. Moreover, research indicates that SGLT2 inhibitors improve neural plasticity and cerebral glucose metabolism, potentially benefiting cognitive function and memory [102]. Their ability to cross the blood–brain barrier (BBB) and brain parenchyma provides insights into their impact on neurovascular integrity, as evidenced by diabetic mouse models showing restoration of neurotrophic levels and control of neuroinflammatory pathways [103].

The latest findings demonstrate that EMPA possesses anti-inflammatory effects in activated microglia, diminishing the expression of pro-inflammatory mediators like IL-6, TNF, and IL-1β, which are associated with neurodegenerative diseases [104]. DAPA and CANA have also demonstrated acetylcholinesterase-inhibiting activity, which is similar to the mechanism of action of drugs in Alzheimer’s disease treatments. Furthermore, it seems that these inhibitors protect the neurovascular unit, avoiding abnormal remodelling in diabetic models [105]. Additionally, SGLT2 inhibitors have a variety of pleiotropic effects, such as preventing brain damage from ischemia/reperfusion, modifying pathways linked to the circadian rhythm, and raising levels of brain-derived neurotrophic factor (BDNF), which is essential for neuronal survival and plasticity.

In addition to its neurological advantages, SGLT2 inhibitors exert considerable cardiovascular effects, especially in alleviating the impact of cerebral small vessel disease (CSVD) [106]. According to studies, SGLT2 inhibition improves white matter integrity and decreases perivascular space expansion while lowering the risk of small vessel stroke and deep cerebral microbleeds. Moreover, these inhibitors facilitate vascular homeostasis by regulating cholesterol metabolism, alleviating endothelial dysfunction, and improving mitochondrial function. Their capacity to diminish inflammatory signaling and oxidative stress is vital for cardiovascular protection, alleviating risks linked to atherosclerosis, heart failure, and chronic kidney disease [107].

The expanding research necessitates further exploration of the CNS-specific and cardiovascular processes of SGLT2 inhibition, facilitating the development of innovative treatment approaches aimed at neurodegeneration, cognitive decline, and vascular health.

## 9. Summary

There has been a significant breakthrough in the treatment of type 2 diabetes with the introduction of SGLT2 inhibitors, often known as flozins [108]. These medications have effects that go well beyond lowering blood glucose levels. These compounds work by inhibiting sodium-glucose cotransporters type 2 in the kidneys, which results in a rise in the amount of glucose that is excreted in the urine. This, in turn, has the effect of reducing blood glucose levels [109]. This mechanism is essential for the effective management of type 2 diabetes. On the other hand, the multifunctional potential of SGLT2 inhibitors deserves particular attention. In patients with heart failure, they reduce circulating blood volume and blood pressure, thereby relieving cardiac workload and improving function [110]. Additionally, they promote a metabolic shift toward more efficient energy substrates, supporting myocardial recovery and reducing the risk of hospitalization [111]. Because of their anti-inflammatory properties, these inhibitors are effective in the treatment of ACS [112]. They also lower the risk of thrombosis and improve endothelial function, both of which are essential for minimizing cardiovascular problems and ensuring that blood flow is maintained properly. Glycemic management, weight loss, and blood pressure reduction are some of the therapeutic advantages of SGLT2 inhibitors. These benefits are especially helpful for patients with hypertension and obesity [113]. Despite these benefits are there, further study is required to fully comprehend their long-term consequences, interactions with other treatments [114], and the influence they have on health, particularly in instances when diabetes is present in conjunction with heart failure or atrial fibrillation [115]. Emerging dual SGLT1 and SGLT2 inhibitors may provide new treatment opportunities, maybe leading to an improvement in the quality of life of patients and a reduction in the risks associated with cardiovascular disease [116]. Owing to their multifunctional properties, these compounds represent a promising class of therapeutics. They pave the way for innovative treatment strategies with the potential to significantly improve patient outcomes and reduce cardiovascular risk.

## Figures and Tables

**Table 1:** Comparison of the basic mechanisms of action of SGLT2 inhibitors in three different disease entities: diabetes, heart failure, and acute coronary syndrome (ACS).

Aspect	Diabetes	Heart Failure	Acute Coronary Syndrome (ACS)
**Basic Mechanism of Action**	Inhibits renal glucose reabsorption Increases urinary glucose excretion Lowers blood glucose	Reduces blood volume and pressure Shifts cardiac metabolism to ketones Improves myocardial efficiency	Reduces oxidative stress and inflammation Enhances endothelial function Improves coronary perfusion
**Similarities in Action**	Lowers glucose and BP Reduces cardiac load Improves endothelial function	Reduces glucose, BP, and volume Anti-inflammatory effects Enhances cardiomyocyte function	Lowers BP and oxidative stress Improves endothelial reactivity Reduces thrombosis risk
**Differences in Action**	Focus on glycemic control Prevents diabetic complications	Focus on hemodynamic relief Improves cardiac remodeling and function	Focus on vascular protection Enhances reperfusion and reduces infarct size
**Therapeutic Synergy**	Reduces CV risk in comorbid HF or ACS Improves metabolic profile	Benefits patients with diabetes and HF Reduces hospitalization and mortality	Enhances outcomes in diabetic ACS Reduces inflammation and vascular events
**Research Perspectives**	Long-term CV outcomes Weight loss and metabolic benefits	Energy metabolism and fibrosis Drug interactions in HF	Endothelial repair Inflammation and thrombosis prevention

## Data Availability

No new data were created or analyzed in this study. Data sharing is not applicable.
